# Intuition as Emergence: Bridging Psychology, Philosophy and Organizational Science

**DOI:** 10.3389/fpsyg.2021.787428

**Published:** 2022-02-03

**Authors:** Paola Adinolfi, Francesca Loia

**Affiliations:** ^1^Department of Management and Innovation Systems, University of Salerno, Fisciano, Italy; ^2^Department of Economics, Management and Institutions, Federico II University of Naples, Naples, Italy

**Keywords:** strategic decision-making, intuition, emergence, complexity science, cross-disciplinary approach

## Abstract

Accelerating environmental uncertainty and the need to cope with increasingly complex market and social demands, combine to create high value for the intuitive approach to decision-making at the strategic level. Research on intuition suffers from marked fragmentation, due to the existence of disciplinary silos based on diverse, apparently irreconcilable, ontological and epistemological assumptions. Not surprisingly, there is no integrated interdisciplinary framework suitable for a rich account of intuition, contemplating how affect and cognition intertwine in the intuitive process, and how intuition scales up from the individual to collective decision-making. This study contributes to the construction of a broad conceptual framework, suitable for a multi-level account of intuition and for a fruitful dialogue with distant research areas. It critically discusses two mainstream conceptualizations of intuition which claim to be grounded in a cross-disciplinary consensus. Drawing on the complexity paradigm, it then proposes a conceptualization of intuition as emergence. Finally, it explores the theoretical and practical implications.

## Introduction

*“The real voyage of discovery consists not in seeing new landscapes, but in having new eyes” M. Proust*

Accelerating environmental uncertainty and the need to cope with increasingly complex market and social demand, combine to create high value for the intuitive approach to decision-making at the strategic level. Nevertheless, despite the growing relevance intuition has in the context of top management decision-making, the topic is still under-investigated in terms of levels of analysis and degree of integration/interdisciplinarity.

First of all, research on individual intuition suffers from a marked fragmentation, with few interactions between groups of researchers and limited cross-disciplinary knowledge accumulation (Hodgkinson and Sadler-Smith, 2018). One reason for such fragmentation is the lack of synergies between scholars from different disciplines and, within disciplines, from different research arenas, characterized by disciplinary silos based on diverse, apparently irreconcilable, ontological and epistemological assumptions.

Advances in research on cognition, particularly the advent of bounded rationality and the increasing relevance of the affective dimension, along with the methodological developments taking place in the field of neurosciences, intensify the need for a cross-fertilization between management and psychology. An example can be seen from the Upper Echelons Theory (UET) in its first conceptualization (Hambrick and Mason, 1984) as well as in its later co-evolutionary interpretation by Abatecola and Cristofaro, 2020, who review UET in a newfangled way. The use among upper echelon scholars of demographic characteristics of top managers as proxies for psychological ones (Finkelstein et al., 2009) is emblematic of the importance of integrating management with psychology. In view of the empirical evidence demonstrating how important psychological characteristic are (Miller et al., 1998; Samba et al., 2018), researchers find it easier to observe demographic characteristics (which can be easily measured from archival data) and draw inferences about cognitive characteristics. Recent empirical evidence suggests that such practices can lead to biased conclusions, given that demographic characteristics may not be a proxy of psychological characteristics, thus pointing to the need to integrate management studies with the most advanced psychological research acquisitions and methodologies.

As recommended by Cristofaro (2017, p. 185), a crucial challenge is to design cross-disciplinary studies which merge different approaches and provide a broader lens than those of the recent past, facing the decision-making issues from a holistic perspective.

There have been a number of attempts to develop a conceptualization of intuitive decision-making which could be testable across disciplines. The first significant attempt was Sinclair and Ashkanasy’s (2005), which drew on an extensive inter-disciplinary literature review including management, psychology and neuroscience. The two authors defined intuition as “a non-sequential information processing mode, which comprises both cognitive and affective elements and results in direct knowing without any use of conscious reasoning” (Sinclair and Ashkanasy, 2005, p. 7). A couple of years later Dane and Pratt, on the basis of a comprehensive review carried out within the field of management, proposed a quite similar conceptualization of intuition—“affectively charged judgments that arise through rapid, non-conscious and holistic associations” (2007, p. 40)—that incorporates research studies from management, psychology and philosophy. This definition has been also followed by psychological scholars (Hodgkinson et al., 2008; Hogarth, 2010; Gore and Sadler-Smith, 2011). There have been other valuable reviews of intuition by Blume and Covin (2011) and Baldacchino et al. (2015), but their focus is limited to a specific of intuition, entrepreneurial intuition, and their construct consolidates the consensual elements of intuition proposed by Dane and Pratt (2007). For example, Baldacchino et al. (2015), starting from Dane and Pratt’s conceptualization, propose an additional element of the conceptualization of intuition, namely domain-specific experience and expertise.

Despite the fact that Dane and Pratt (2007) present their construct as a relatively non-contentious deployment of conjectures grounded in an interdisciplinary consensus, their attempts to formulate a conceptualization of intuition which is accepted across distant disciplines do not appear to have increased conceptual integration and knowledge accumulation. In particular, despite Dane and Pratt’s claim “to bridge works in psychology, philosophy and management” (2007, p. 36), there are no quotations from philosophy scholars in about 1977 works citing their 2007 article. On the whole, their cross-disciplinary conceptualization has not led to the development of a grand theory of intuition and, after more than 10 years, the matter is far from closed. Other authors who cross-disciplinarily reviewed literature on intuition, such as Carter et al. (2017), have had a limited impact to date, as proved by the limited number of quotations (22), if compared with Dane and Pratt’s quotations (1977) and Sinclair and Ashkanasy (532).

In this framework, the present paper critically discusses Sinclair and Ashkanasy’s construct, which has been the first significant attempt to develop a cross-disciplinary conceptualization of intuition, and Dane and Pratt’s article, which has been the most influential article related to the intuition in management literature and other disciplinary fields. Dane and Pratt’s article is regarded as the most comprehensive and most referenced definition of intuition (Okoli et al., 2021). Our paper analyzes the proposed intuition’s attributes and assesses the degree of cross-disciplinary agreement around them, with the purpose of enhancing the intuition construct in a way that facilitates inter-disciplinary dialogue. In the light of such analysis, a definition of intuition is proposed, as “knowing that emerges out of self-organizing holistic associations,” and the reasons for this emergentist account of intuition are explained in the discussion section. In the conclusive sections theoretical and practical implications are outlined as regards UET, the interaction between intuitive and deliberative processing modes, the conditions for intuition’s effectiveness, the relationship with artificial intelligence, the possibility to the escalation from individual to collective decision-making. The limitations of the study as well as the avenues for future research are also highlighted.

Space permits only a brief overview of the huge literature on decision-making (the whole review is available on request), but it is sufficient to question the mainstream conjectures. We focus on the three domains which are considered in the examined articles—philosophy, psychology and organizational science/management—and identify conceptual intersections around which potential cross-disciplinary agreement on the definition of intuition can be envisaged. Drawing on complexity science, a framework to accommodate both positions is then hypothesized, which conceptualizes intuition as emergence. Finally, some implications are explored.

## Methods

In line with the aim of constructing an encompassing conceptualization of intuition, both Sinclair and Ashkanasy and Dane and Pratt propose a definition that seeks conceptual agreement across different disciplines: Sinclair and Ashkanasy (2005) include behavioral sciences (neuroscience and psychology) besides management, whilst for Dane and Pratt (2007, p. 39) the central characteristics of intuition are identified “based on their commonality to definitions across philosophy, psychology and management”. In addition to their commonality, the central characteristics also appear to be the most “core” features (p. 39), and account not just for what intuition is but also how it “differs from other decision-making approaches” (p. 40).

So, a critical revision of the mainstream definition of intuition implies checking whether such definition correctly sets out the “core” characteristics of intuition, and whether it adequately differentiates the concept from others, such as rationality, instincts, insight, and learning. In formal terms, this implies that the attributes (A) identified to define intuition (I), are necessary and sufficient conditions for inferring it; more precisely: each of the attributes (Ai) is a necessary condition for (I), and all the attributes (Ai…n) are, together, a sufficient condition for (I).

Such an assessment must be inter-disciplinary, since both Sinclair and Ashkanasy and Dane and Pratt target a strong inter-disciplinary agreement. Given the distance between the disciplines, various degrees of cross-disciplinary agreement are contemplated in this paper, as shown in Table 1, so that an agreement field is traced, showing the degree to which the different perspectives may experience interaction and build on common elements together synergistically. The table shows the following dimensions:

**TABLE 1:** Levels of cross-disciplinary agreement.

Degrees of cross-disciplinary agreement	Description
Strong agreement	The attributes identified are necessary and sufficient conditions for intuition in the most prominent streams of research within the selected disciplinary domains
Weak agreement	There is at least one prominent stream of research within each disciplinary domain in which the attributes identified are not necessary and sufficient for intuition
Potential agreement	There are epistemological elements from which to infer that the attributes identified are necessary and sufficient conditions for intuition in at least one stream of research in each disciplinary domain
No agreement	The attributes identified are not necessary and sufficient conditions for intuition in the most prominent streams of research within the selected disciplinary domains

*Authors’ elaboration.*

(a) Strong agreement: The attributes identified are necessary and sufficient conditions for intuition in the most prominent streams of research within the selected disciplinary domains;
(b) Weak agreement: There is at least one prominent stream of research in one disciplinary domain in which the attributes identified are not necessary and sufficient for intuition;
(c) Potential agreement: There are epistemological elements from which to infer that the attributes identified may be considered necessary and sufficient conditions for intuition, since they are not in contrast with the in-depth assumptions of at least one stream of research. This category allows possible cross-fertilizations to be highlighted in cases in which terms are used differently in different contexts or are not clearly defined. The alternative would be to acknowledge that no dialogue is possible;
(d) No agreement: The attributes identified are not necessary and sufficient conditions for intuition in the most prominent streams of research within the selected disciplinary domains.

It could have been sufficient to analyze Sinclair and Ashkanasy’s construct, which is antecedent and similar to that of Dane and Pratt’s, but Dane and Pratt’s construct has been increasingly accepted in the field of management as the consensual definition; it is regarded as the most comprehensive and most referenced definition of intuition (Okoli et al., 2021): for example, Akinci and Sadler-Smith (2012, p. 115) wrote that, despite several studies flourishing throughout the years, Dane and Pratt’s is the only “comprehensive, integrated account and testable research propositions”. So the article has formed the basis of a number of subsequent influential literature reviews, conceptual contributions, and methodological and empirical works; drawing from it, scholars have differentiated intuition from related constructs (Hodgkinson et al., 2008, 2009a,b; Sadler-Smith, 2010, 2015, 2016; Salas et al., 2010; Blume and Covin, 2011; Akinci and Sadler-Smith, 2012, 2013, 2019, 2020; Baldacchino, 2013, 2019; Baldacchino et al., 2015; Healey et al., 2015; Pratt and Crosina, 2016; Calabretta et al., 2017; Okoli et al., 2021; Sadler-Smith et al., 2021). In addition, the article has spread over psychology and neuroscience (Hodgkinson et al., 2008; Hogarth, 2010; Gore and Sadler-Smith, 2011; Hodgkinson and Healey, 2011; Ben-Soussan et al., 2020; Keck and Tang, 2020; Korteling and Toet, 2020; Li et al., 2020; Stephens et al., 2020; West et al., 2020; Muñoz-Cobos and Postigo-Zegarra, 2021; Reynolds et al., 2021; Yu et al., 2021; Zhang et al., 2021), as well as distant disciplinary domains, such as medicine and health sciences (Glatzer et al., 2020; Cameron and Singh, 2021; Chlupsa et al., 2021) or engineering and design (Cash and Maier, 2021; de Rooij et al., 2021; Paige et al., 2021; Park et al., 2021).

In addition, compared to Sinclair and Ashkanasy (2005), Dane and Pratt’s article embraces a wider interdisciplinary range which includes philosophy. This is another reason to focus attention on Dane and Pratt’s article, considering that the concept of intuition (as well as many other lemmas used in managerial or psychological disciplines) has a philosophical origin, and, consequently, cross-fertilization with philosophical studies on intuition is beneficial to allow a better understanding of the historical use as well as the variety of contemporary uses of the concept of intuition.

## Mapping the Territory: Critical Review of Intuition’s Attributes

*“The whole object of travel is not to set foot on foreign land; it is at last to set foot on one’s own country as a foreign land” G. K. Chesterton*

As mentioned in the introduction, in 2005 Sinclair and Ashkanasy proposed an overarching definition of intuition to incorporate research studies from management, psychology and neuroscience: “a non-sequential information processing mode, which comprises both cognitive and affective elements and results in direct knowing without any use of conscious reasoning” (p. 7). A couple of years later, Dane and Pratt (2007) proposed a quite similar conceptualization of intuition—“affectively charged judgments that arise through rapid, non-conscious and holistic associations” (2007, p. 40) (p. 40)—that drew on an extensive inter-disciplinary literature review including management, psychology and philosophy. In this section, we critically evaluate the widely accepted definitions of intuition by Sinclair and Ashkanasy (2005) and Dane and Pratt (2007), with a focus on the latter. We draw on the fields of philosophy, psychology and management to identify which elements of these definitions are necessary and sufficient for a parsimonious conceptualization of intuition, on the basis of cross-disciplinary consensus (weak, strong and potential agreement, as defined in the methodological section).

### Holistically Associative

According to Dane and Pratt, intuition involves a process in which disparate elements are associated with one another (2007, p. 37). Such associations are holistic, referring to whole structures or patterns, which are simplified representations of reality, implying analogical representation of information. Before Dane and Pratt, Sinclair and Ashkanasy had already included in the construct of intuition the attribute of being non-sequential/holistic, referring to information processing. In both contributions, the difference between intuitive and (what is called) rational thinking is related to the difference in type of information processing: opposed to the linear-sequential processing typical of rational thinking, the holistic-associative type is consistent with “intuitive” thinking (Barrafrem and Hausfeld, 2020; Alaybek et al., 2021; Zhang et al., 2021), so rationality vs. intuition means Fregean vs. analogical language; sequential vs. simultaneous relationships; details vs. big picture; central vision vs. peripheral vision; gaze vs. glance; text vs. context; focused attention vs. broad, open, mindful alertness.

Contrary to Dane and Pratt’s assertions, our review of philosophical literature shows that the attribute of being holistically associative is not present in classical accounts of intuition, such as Descartes (1637-1998) and post-Carthusian philosophy (Spinoza, 1677–2007; Locke et al., 1690-2006), as well as the 1900s phenomenological philosophy of intuition (Husserl, 1900/01-2015). In all these, intuition does not extract one possible representation from an infinite range of possibilities but rather grasps an objective characterization of the real problem setting. An holistically associative view of intuition can be more appropriately related to emergentist or pragmatist philosophers, although these philosophical streams of thought may not expressly mention intuition.

The holistic-associative account of intuition is acknowledged by psychology scholars as well as management scholars (Agor, 1986; Dreyfus and Dreyfus, 1986; Kihlstrom, 1987; Simon, 1987; Prietula and Simon, 1989; Shapiro and Spence, 1997; Kahneman and Tversky, 2000; Betsch and Glöckner, 2010; Glöckner and Witteman, 2010). It forms the basis of spreading activation in neurological models of insight—the “aha,” eureka moment of insight occurs when particular ideas fall into place, as concepts stored disparately in the brain are conjoined and the solution literally pops into consciousness. Associative reasoning represents an important feature of Type-1 processing, according to the dual-process theories of cognition.

Dual-process theories have increasingly developed in various strands of psychology research since the beginning of the new century as an attempt to capture the duality of human cognition, which is accomplished in two types of process: intuitive (Type 1), namely unconscious, rapid, automatic, and high capacity processes; and reflective (Type 2), namely conscious, slow and deliberative processes (Evans, 2010). These theories have gained legitimacy in social psychology and cognitive science over the last two decades and have been corroborated by neurosciences (Lieberman, 2007).

In the last few years, research on decision-making has tended to move progressively toward models of decision-making that acknowledge, rather than reduce, the complexity of the world, ultimately challenging the traditional hierarchical and dualistic way of thinking by proposing frameworks that comprehend and reconcile antinomies: initially there was a prevalence of the so-called *default-interventionist* of dual theories of decision-making, which assume that “a basic default position in human processing is to rely on less costly Type 1 (intuitive) processes in order to conserve the scarce cognitive resources required for Type 2 (analytical) processes, deploying the latter only as and when essential” (Hodgkinson and Sadler-Smith, 2018, p. 11); these theories have been progressively supplanted by *parallel-competitive* theories, that assume that Type 1 and Type 2 processes operate in parallel, and, in the event “of conflicts between them, they literally compete for the control of thinking and behavior” (Hodgkinson and Sadler-Smith, 2018, p. 11).

In all their various formulations, these theories regard holistic-associative as an attribute of intuitive processing. On the whole, it can be said that there is strong agreement across management and psychology that holism is a necessary attribute of intuition, but no agreement with philosophers. Therefore, Dane and Pratt’s conjecture of strong cross-disciplinary agreement must be refuted, while Sinclair and Ashkanasy’s conjecture, which includes only management and behavioral disciplines, is acceptable.

### Affectively Charged

Dane and Pratt (2007, p. 39) state that intuitive judgments “may be thought of as affective because they are detached from rationality.” So, the attribute “affective” is used because intuitive judgments appear detached from cold rationality. This argument is syllogistic: (a) intuition is detached from rationality; (b) rationality is not affectively charged; (c) intuition is affectively charged. This syllogism appears incorrect: not only does the premise (a + b) not logically imply the conclusion (c) in that “detached from” does not mean “opposed to,” but the premise is not proved at all by the two authors, as their definition of rationality does not imply necessarily unemotionality.

To support their assertion, Dane and Pratt state (p.40) that “the coupling of affect and intuition has a very long intellectual history.” They mention a generic “common divide in philosophy,” which contrasts rationality/head with intuition/heart. This can be vigorously rejected. While Spinoza’s concept of *amor intellectualis* (1677–2007) corroborates the idea of affective rationality, the intuition-affect association is quite extraneous to rationalistic philosophical tradition: it is present neither in the classical (Aristotle) nor in the rationalist accounts of intuition (Descartes, 1637-1998; Locke et al., 1690-2006). These remarkable philosophers regard intuition as the vehicle of apprehension of first principles and self-evident understandings that grounds all knowledge, a necessary condition for deductive thinking, since the steps in a chain of demonstrative reasoning are “intuitively grasped.” Intuition is thus not in contrast to rational thinking but is the very basis on which this form of deliberative reasoning proceeds. The notion of intuition as integral to rational thinking can also be found, with all the epistemic differences, in the empiricist tradition (Bacon, 1620-1992; Locke et al., 1690-2006; Hume, 1738/40-2001), in Kant (1781-2013)) and the idealistic post-Kantian revivalin the twentieth century European philosophy (Wittgenstein, 1914-1953). It can be found, too, in the philosophy of mathematics (Russell and Whitehead, 1910; Whitehead and Russell, 1912; Hodgkinson et al., 2008; Godel, 1964/2011): intuition is considered as the basis for mathematical understanding, fundamental to rule-based reasoning and the manipulation of symbols.

As regards psychological and managerial literature, Dane and Pratt (2007, pp. 38–39) report several empirical findings from the 1980s and 1990s to support their thesis. They quote Burke and Miller’s (1999) statement that “managers *often* view affect as an important input to intuition” and report Hogarth’s (2001) assertion that “emotion and affect *can*, therefore, be important inputs to intuitive thought.” Moreover, they refer to Bastick (1982) and Epstein (1994) to assert that emotions and affect *may* often also play a role in the intuition process itself. In light of this empirical evidence, the two authors conclude that “research suggests that affect is associated both with the intuiting process and with intuition as an outcome” (p. 39).

This conclusion is not rigorously demonstrated: the quoted studies do not prove that affect is a necessary condition for intuition since the terms used (*often*, *can*, *may*; italics added) imply possibility, not necessity. Surprisingly, Dane and Pratt themselves (2007, p. 35) report a list of definitions of intuition, of which no one includes affect or similia.

Management studies of the same period (Behling and Eckel, 1991; Isenberg, 1991; Brockmann and Anthony, 1998; Gavetti and Levinthal, 2000) also assert that affect is not a necessary condition for intuition, too. A number of studies have identified alternative forms of intuition variously incorporating hot and/or cold processes. For example, the notion of expert-based intuition does not consider affect as an essential defining feature. Recently, Cristofaro (2019; 2020; 2021) analyses in depth the role of affect in management decisions, and, in the light of a systematic literature review, synthesizes the academic research into a comprehensive framework, in which affect is an element that interacts with bounded rationality, and is always present in both intuitive and non-intuitive processes; because of that, affect cannot be a feature of just intuitive processes, rather featuring the overall management decisions in organizations (Cristofaro, 2019).

There are, in addition, a number of psychology studies of the same period that stress the cognitive nature of intuition as opposed to the affective nature of intuition (Crossan et al., 1999). Examples include the experimental psychology studies focused on the shortcomings of intuition in comparison with rationality, summarized by Nisbett and Ross (1980) and Tversky and Shafir (1992); even the two meta-analyses quoted by Dane and Pratt themselves (Osbeck, 1999, 2001) assert that affect is not an attribute of intuition.

Compared to Dane and Pratt’s article, the study by Sinclair and Ashkanasy (a couple of years before) appears more balanced. The two authors propose an articulated, threefold account of the relationship between intuition and affect, where the latter may (but does not necessarily) play important roles as regards intuition. “First, in the pre-intuitive stage, affect (either trait or state) may […] act as a determinant […] or a moderator […] of intuition. Second, during the intuitive process, some people tend to use affect as their preferred mode of reception […]. In this case, affect becomes a component of the intuition construct itself. Finally, in the evaluation stage, individuals experience confirmation of the “genuine” nature of intuition through a specific feeling, such as relief or certitude […]. We see this as an accompanying symptom of the intuitive process” (Sinclair and Ashkanasy, 2005, p. 358).

In conclusion, drawing on the logical considerations and empirical evidence reported above, only weak agreement across management and psychology can be proved, and no agreement at all in philosophy, so we can reject Dane and Pratt’s statement that affect is a necessary condition for intuition. Obviously, this does not imply that affect cannot be an important correlate of intuition, and even if it may not be a component of the cognitive process that leads to intuition, it may well be a trigger or an accompanying symptom of it, as highlighted by Sinclair and Ashkanasy (2005). This would explain similarities across disciplines.

### Unconscious, Sub-Conscious, Pre-conscious, Supra-Conscious

According to Dane and Pratt, in philosophy, the unconscious account of intuition is central. To support their arguments, they quote Osbeck (2001, p. 123), a scholar of psychology, who writes that intuition from a philosophical perspective involves direct apprehension that is “not mediated by other reasoning or representation” (Dane and Pratt, 2007, p. 37). Yet Osbeck does not mean that intuition, from a philosophical perspective, is unconscious, just “not mediated by reason.”

According to Dane and Pratt, in philosophy the unconscious account of intuition is central. To support their arguments, they quote Osbeck (2001, p. 123), a scholar of psychology, who writes that intuition from a philosophical perspective, involves direct apprehension that is “not mediated by other reasoning or representation” (Dane and Pratt, 2007, p. 37). Yet Osbeck does not mean that intuition, from a philosophical perspective, is unconscious, just “not mediated by reason.” This is confirmed by the fact that her words are followed by a philosophical account of intuition as “seeing” essential natures and first principles with the intellect: in her view, the best means of expressing intuition in philosophical literature is the “vision metaphor,” an ancient concept maintained in contemporary discourse: it can be traced at least to Pythagoras’ appeal to super-sensuous vision and to Plato’s “eye of the soul,” and was extended by several later scholars, such as Agostino, Descartes, Spinoza. As regards specifically the conceptual division conscious/unconscious cognition, Osbeck (2001, p. 244) observes: “this is not a relevant distinction as concerns intuition, according to the means by which this notion has been understood historically.”

So, the unconscious account of intuition is not associated to the vision metaphor which is predominant in philosophy.

The concept of unconscious originated from psychological studies (Jung, 1933) and spread in management literature. However, debate still continues over how to define consciousness (Churchland, 2002) and how to establish unconscious processing, given the difficulty of separating it from conscious (Overgaard et al., 2006; Sandberg et al., 2010); in particular it remains controversial whether unconscious processes involve a rigorous all-or-none mechanism or lie on a continuum. Miller and Schwarz (2014) argue that conscious awareness of intuitive decisions builds gradually and they deny its all-or-none character. If consciousness is a fuzzy variable along an unconscious-conscious continuum, then the problem emerges of where to demarcate intuition and insight.

Management scholars appear to be aware of the debate concerning the complications of separating controlled and automatic processes, and the extent to which intuition is a conscious and/or non-conscious process. Different authors have used different labels to describe different levels of sophistication for processes that are not conscious (see Dane and Pratt, 2007): “preconscious” (e.g., Crossan and Berdrow, 2003), “subconscious” (e.g., Henderson, 1977; Khatri and Ng, 2000; Raidl and Lubart, 2001; Miller and Ireland, 2005), “unconscious” (e.g., Jung, 1933; Reber, 1992; Slaughter, 1996) and “non-conscious” (Simon, 1987; Agor, 1989; Epstein, 1994; Shapiro and Spence, 1997; Lieberman, 2000; Hogarth, 2001). Dane and Pratt use the term “non-conscious” to encompass all levels beyond an individual’s consciousness. This is not original, since Sinclair and Ashkanasy in 2005 used the term non-conscious, drawing on Wally and Baum’s (1997) portrait of intuition. However, there is contradictory evidence about that. For example, Strack and Deutsch (2004) provide evidence that reflective and reflexive cognitive processes co-occur, concluding, in line with other scholars (Dijksterhuis, 2004; Ham and Van den Bos, 2011), that conscious thinking is a combination of conscious and unconscious processes.

In conclusion, the term “non-conscious,” used by Sinclair and Ashkanasy and then adopted by Dane and Pratt, appears more appropriate than other nuanced concepts such as “unconscious,” “pre-conscious,” “sub-conscious” and “supra-conscious”, but it is indeed called into question by a number of empirical studies. So, Dane and Pratt’s conjecture that there is strong interdisciplinary agreement about the attribute “non-conscious” as a necessary condition for intuition should be rejected, since only weak agreement can be envisioned across management and psychology, and no agreement with philosophy.

### Fast and Direct

The characteristic of intuitive synthesis that has garnered the most interest among both scholars and practitioners is the speed at which it leads to choice (Patel et al., 2019). For upper echelons in particular, the speed of intuitive synthesis has great appeal and is seen as the primary driver for developing, promoting and using intuition at work (Agor, 1986; Burke and Miller, 1999; Khatri and Ng, 2000; Klein, 2003). This is indeed the element which differentiates Dane and Pratt’s (2007) construct from Sinclair and Ashkanasy’s (2005) construct: namely the attribute “fast” instead of “direct.” To corroborate the conjecture of strong interdisciplinary agreement on the attribute “fast,” Dane and Pratt (2007, p. 34) quote two philosophers, Wild (1938) and Rorty (1967), making indeed a big philosophical mistake: both philosophers use the term immediate “immediate apprehension”—but not in the temporal sense of rapidity, rather, in the logical sense of “without any mediation.” In addition, Rorty reports this definition as classical philosophers’ definition, not as his own, and follows Peirce’s (1970) critique of intuition, according to which intuition is never immediate, it is always mediated.

While directness of intuition is central to Western philosophers, the idea of fastness as a necessary characteristic of intuition, distinguishing it from rational thinking, is not present in most philosophical accounts of intuition, such as Plato’s *nous*, Aristotle’s *noesis* (Zalta, 2004), Descartes (1637-1998) *intuitus mentis*, Spinoza’s (1677–2007) *scientia visionis* or Husserl’s 1900/01-2015 phenomenological intuition.

As regards the psychological and managerial literature, lengthy intuitive processes are evidenced in notable empirical studies. Besides Hogarth’s (2001) study, quoted by Dane and Pratt, there are studies on the “unconscious thought effect” (Dijksterhuis, 2004; Dijksterhuis and Nordgren, 2006), suggesting that extended processing time may precede some forms of intuition, such as creative intuition and problem-solving intuition. These studies show that, in complex intuitive tasks, deciding after unconscious thinking for a couple of minutes produces superior performance compared to an immediate decision. The superiority of long-lasting over instantaneous intuition seriously undermines the idea of fastness as integral to the concept of intuition, suggesting that a time-consuming, non-conscious elaboration process may occur before intuition emerges.

Dane and Pratt solve this contradiction by recurring to the concept of incubation (Gilhooly, 2016) and distinguishing between “intuition,” the territory of pure unconsciousness, and “insight,” a concept used in psychology (Zander et al., 2016). They define insight as “conscious recognition of the logical connections” supporting a particular solution, and articulate it in two discrete steps (analytical and intuitive); yet they do not demonstrate this, and, indeed, it is hard to demonstrate, due to the ubiquitous continuity of mental activity (Chia, 1998). Dane and Pratt (2009) themselves, in a later article (2009, p. 27), warn: “researchers must also be cautious to avoid dismissing certain forms of cognition (e.g., processes that involve incubation) as definitely non-intuitive, as doing so might minimize the power and richness of the intuition constructs”; they recognize that not only insight but also creative intuition appears to be preceded by an incubation period. So, creative intuition would be an example of slow intuition. In addition, examples of slow intuition are quite frequent in collective decision-making.

Indeed, rather than economies of time, it can be demonstrated (going back to Sloman, 1971), that economies of effort stem from holistically associative intuitive processes, which are based on analogical processing (these have to do with directness).

In conclusion, contrary to Dane and Pratt’s assertion, there is no strong agreement within and between the three disciplines (and even within Dane and Pratt’s scientific production itself) that the attribute fast is a necessary condition for intuition; there is only weak agreement across psychology and management. However, rather than demonstrating economies of time, it is possible to demonstrate economies of effort stemming from the analogical, synthetic nature of intuitive processing.

## Overview: Searching for Intersections

*“It is not down in any map: true places never are.” H. Melville*

The results of the analysis are synthesized in Table 2, which shows, for each proposed attribute, the sources that disagree that it is a necessary condition for intuition. Despite what declared by Dane and Pratt, for none of the attributes they identified there is full cross-disciplinary consensus: the attribute “affectively charged” is put in question by significant streams of research in the three disciplines, the attributes “non-conscious” and “fast” are questioned by significant streams of behavioral research, besides philosophy, while the attribute “holistically associative” is not in line with philosophical literature. On the whole, a strong agreement can be advocated only as regards management studies, while there is a weak agreement with psychology studies, and no intersection with philosophy: in their effort to compress the notion of intuition into mainstream psychological and managerial categories, Dane and Pratt have misinterpreted philosophical research on intuition.

**TABLE 2:** Sources that disagree with the proposed attributes of intuition.

Necessary and sufficient conditions for intuition	Management	Psychology	Philosophy
Holistically associative	–	–	**Disagree** (Descartes, 1637-1998; Spinoza, 1677–2007; Leibniz, 1720-2001; Husserl, 1900/01-2015)
Affectively charged	**Disagree** (Behling and Eckel, 1991; Isenberg, 1991; Brockmann and Anthony, 1998; Gavetti and Levinthal, 2000)	**Disagree** (Nisbett and Ross, 1980; Tversky and Shafir, 1992; Crossan et al., 1999; Osbeck, 1999, 2001; Kahneman and Tversky, 2000)	**Disagree** Aristotle (Bacon, 1620-1992; Descartes, 1637-1998; Locke et al., 1690-2006; Leibniz, 1720-2001, Hume, 1738/40-2001; Kant, 1781-2013), Zalta, 2004), early (Russell and Whitehead, 1910, 1912; Wittgenstein, 1914-1953; Hilbert, 1925-1983; Godel, 1964/2011; Hodgkinson et al., 2008)
Non-conscious	–	**Disagree** (Osbeck, 1999, 2001; Dijksterhuis, 2004; Strack and Deutsch, 2004; Overgaard et al., 2006; Sandberg et al., 2010; Ham and Van den Bos, 2011; Miller and Schwarz, 2014)	**Disagree** Pythagoras, Plato (Agostino, 1387-2002, Descartes, 1637-1998; Spinoza, 1677–2007; Zalta, 2004)
Fast	–	**Disagree** (Hogarth, 2001; Dijksterhuis, 2004; Dijksterhuis and Nordgren, 2006)	**Disagree** Plato, Aristotle (Descartes, 1637-1998; Spinoza, 1677–2007; Husserl, 1900/01-2015; Zalta, 2004)

*Authors’ elaboration.*

Strikingly, the conceptualization provided by Sinclair and Ashkanasy 2 years before Dane and Pratt appears more appropriate: the attribute “direct” in place of “fast” appears more in line with philosophical accounts of intuition, albeit Sinclair and Ashkanasy never claim to bridge management and philosophy, while the term “knowing,” conveying the idea of both a process and its outcome, appears more appropriate than the term “judgment” used by Dane and Pratt, which only focuses on the conclusive outcome, and, moreover, implies careful reflection. Furthermore, Sinclair and Ashkanasy provide a more detailed account of affect, acknowledging that it is not always an integral component of the intuitive process: in some cases, it may be involved in the antecedent or subsequent processes.

Another weakness of Dane and Pratt is that, in their attempt to provide an encompassing account of intuition, they mistakenly treat intuition’s attributes as necessarily co-occurring. This has been widely accepted amongst scholars (Calabretta et al., 2017) and has inspired a number of empirical studies. Readers tend to align all of them, so that intuitive processing must be hot/non-conscious/holistic/fast, while analytical processing must be cold/conscious/sequential/slow. As a consequence of this, Dane and Pratt have dismissed certain forms of cognition (cold or slow intuition) as definitely non-intuitive, thus missing the opportunity to increase both the richness of their construct and its suitability for interdisciplinary dialogue. Surprisingly, Sinclair and Ashkanasy’s 2005 conceptualization is richer in that it includes cognitive processes involving incubation, or entailing affect not as component of the intuitive process, but as trigger or symptom of it.

In conclusion, contrary to Dane and Pratt’s claim, the four attributes—affective/non-conscious/holistic/fast—are not necessary features of intuition, but are simply correlate features that may occur under specific conditions.

To have an agreed definition of intuition, it would be necessary to identify defining features, namely attributes, that are cross-disciplinarily considered necessary conditions, and, all together, sufficient conditions for intuition. Indeed, managerial and psychological literature, on the one hand, and philosophical literature, on the other, appear incommensurate: they have no common denominator. Nevertheless, to benefit from interdisciplinary dialogue and cross-fertilization, it is still worthwhile searching for potential agreement across the three disciplines. So, we identified two potential intersections.

### An Intuitionist View of Intuition

On the one hand, there is an intersection around the notion of intuition as immediate apprehension, which is meaningful for classical philosophers (called intuitionists) but not corroborated by the mainstream psychological and managerial literature reported by Sinclair and Ashkanasy and by Dane and Pratt. Indeed, such an intuitionist notion has potential for agreement with various types of foundational theories sharing a universalistic and ahistorical viewpoint.

More specifically, as regards management, this foundational view of intuition is epistemologically congruent with early management theories which aimed to base the grounding of knowledge (previously provided by experience) in objective scientific principles. These range from Taylor’s scientific management in its various national declinations, such as German rationalism, Russian Taylorism and French rational administration and technological utopianism, to the more recent neo-Tayloristic developments of management science which have deeply shaped modern society and still do.

All these theories have an epistemological affinity with classic philosophers in being intuitively grasped and presented as truth, absolutely valid and universally applicable in any context, independently from their temporal and geographical collocation. An aspiration to escape temporality and contingency can also be found in normative theories of business ethics such as social contracts theory, stakeholder theory, utility-rights-justice-care, and deontology-utilitarianism.

As regards psychology, the intuitionist view of intuition is congruent with early psychological studies of not only cognitivists but also moderate constructivists, such as Bruner (1986), Harré (1986), and Osbeck (1993), who recognize an (at least partial) ontological independence of reality from social construction.

This cross-disciplinary intersection is only potential, since most of the mentioned management and psychology scholars do not refer to the concept of intuition, but is valuable: “appeal to some versions of direct apprehension has been central to philosophy almost from its inception and the need for this appeal does not disappear in contemporary theory” (Osbeck, 2001, p. 127). Adopting an intuitionist view allows contemporary research on intuition to be aligned with historical philosophical accounts thereof, thereby highlighting its deep historical embeddedness.

### An Anti-intuitionist View of Intuition

On the other hand, there is an intersection around Sinclair and Ashkanasy’s conceptualization of intuition, which is far from the classical philosophical account of intuition, but compatible with an anti-intuitionist epistemology, as expressed by Wittgenstein (1953, 2017)—considered the greatest twentieth-century philosopher (Lackey, 1999)—in what is considered one of the most influential twentieth-century works in cognitive science. This author denies intuition as immediate apprehension and truth as objectively existent. In his view, which we define as anti-intuitionist, truth is not intuited by the mind but is, instead, constructed in localized contexts through linguistic processing, which occurs unconsciously under standard conditions. This automatic process, based on holistic processing, can be assimilated to the notion of intuition proposed by Sinclair and Ashkanasy, involving directness and non-consciousness as likely correlate features. Also the attribute of affect is an expected correlate feature of this conceptualization: if intuition is an act of interpretation linking the world to the intuitor, the full fabric of such an interpretative act is inclusive of feelings and emotions, which are part of the intuitor’s semantic horizon.

As for the intuitionist view of intuition, the intersection around an anti-intuitionist view is only potential, yet it offers great opportunity for enriching the concept of intuition, particularly for contemplating different time spans, several mediating and moderating contextual variables, and multiple levels of analyses.

## Discussion: An Emergent View

*“All journeys have secret destinations of which the traveler is unaware.” M. Buber*

The anti-intuitionist account differs significantly from the intuitionist alternative, as can be highlighted by expressing both in formal and compacted terms.

In the intuitionist view, X has the intuition that Y, merely on the basis of grasping Y; in the anti-intuitionist view, X has the intuition that x, based on holistic extraction of the pattern y from the space of possibilities Y.

These schematized differences can be ascribed to the opposite underlying paradigms, synthesized below.

The intuitionist interpretation of intuition is consistent with a positivist perspective, focused on a phenomenal world irreducibly and unproblematically intuited by a disinterested actor who remains external to what is being intuited and passive (the world is given to the intuitor).

The anti-intuitionist account of intuitive judgments is consistent with a constructivist perspective, where the role of the subject is active, contextually embedded, and time-situated.

In light of the above considerations, two questions emerge. Firstly, does there exist an overarching framework that encompasses the two accounts of intuition, or are the two incommensurable in a Kuhnian sense? Secondly, would such a framework offer new theorizing or would it resemble a 1:1 scale map, which includes everything and provides no additional value? To tackle these questions, an overarching framework is hypothesized, and its implications are evaluated.

### Intuition as Emergence

Intuition could be conceived as emergence—a new property that emerges in a decisional space. This concept concerns the supervenience of a property within a whole system, which cannot be reduced to the properties of the individual parts and cannot be predicted before it manifests itself *de facto*.

An example is sodium chloride. Sodium is a soft, shiny metal, that is inflammable if put in the water. Chlorine is a toxic gas used as a lethal weapon during the First World War. Knowing the properties of these two components, one would not predict that their combination produces a delicious adornment to fried potatoes. The properties of the chemical combination of sodium and chlorine can be predicted after seeing what they *de facto* produce. Moreover, as a characteristic, salty explicates itself in relation to an experiencing subject and cannot be imagined in the absence of such a relationship. This suggests that supervenient properties are not simply intrinsic to the system to which they are attributed but are, instead, essential relational properties (Zhok, 2011). Similarly, intuition emerges from the relationship between the intuiting subject and a set of cues characterizing his/her task/environment: as salty explicates itself in a dynamic relationship with the perceiving subject, intuition explicates itself in a dynamic relationship with the intuitor.

The phenomenon of emergence implies non-linearity: if we admit that relationships between irreducible units produce second-order properties, we can expect discontinuities in the production of effects. Discontinuities and thresholds exist throughout our natural world: not all that happens within a system (cell, organism, etc.) transmits its effects to the superior level; below certain thresholds, nothing passes from one level to that above (which also explains why autonomous entities exist), while, over certain thresholds, a phase transition to a superior order emerges. This also occurs at the infinitesimal level: in quantum theory, energy can be transmitted or adsorbed only discontinuously, by quanta, which implies that not always and not all energetic variations produce effects.

Similar discontinuities might occur at a mental level. It can be hypothesized that the huge volume of cues—as filtered by our perceiving system from the infinite space of possibilities, and combined with the information stored in memory—forms a temporary network (the problem space) which, under external perturbations, is elaborated through iterative associations at a non-conscious level, such that several parallel processes can self-organize without being coordinated by a deliberate system. Drawing on Glöckner and Betsch (2008), we can hypothesize that self-organization is governed by simple order-generating rules, such as consistency: when an association combining several nodes (cues and options) reaches a certain threshold of consistency, intuition emerges as a stable solution which activates the option allowing the threshold to be reached.

In conclusion, the following conceptualization could be proposed: *intuition is knowing that emerges out of self-organizing holistic associations*. In this proposition, the properties of self-organization and emergence are the defining features of intuition. The concept of self-organization replaces the attribute “non-conscious,” allowing the subtleties and difficulties highlighted in the previous paragraphs to be overcome; as observed, it is necessary but not sufficient, since it does not allow instincts and habits to be differentiated. Such differentiation is possible if the property of emergence is added: while in habitual or instinctive processes there is no emergence and no learning (Brown et al., 2020), in intuitive processes, new properties emerge from the interaction of individual cues which are not present in the single parts (leading to the solution of a problem, a creation—i.e., new knowledge).

Besides its philosophical meaning, the term “emergence” conveys the idea of reaching the end of a process, therefore simultaneously conveying the idea of a process and of suddenness, and so it appears more appropriate than “arise,” proposed by Dane and Pratt, evoking mere occurrence.

For the same reason, instead of the term “judgment”—which is a noun—used by Dane and Pratt, the term “knowing” is preferred. Being both a verb and a noun, “knowing” conveys the idea of both a process and its outcome. In this way, the term gives the idea that intuition is never fully accomplished and stable: in fact, it is not only the output of a cognitive process but also one of the myriad cognitive inputs from whose interaction other intuitions will emerge in a seamless cognitive flow.

The attribute “holistic” has been excluded from the proposed conceptualization so as not to violate the fundamental scientific principle of parsimony (Hodgkinson and Sadler-Smith, 2018), since the philosophical concept of emergence entails the idea of holism. For the same reason other properties deriving from the emergent character of intuition need not be reported in the definition, such as unpredictability *ex ante*, situatedness, interactivity or generativity.

Alongside the above parsimonious conceptualization, stripped down to what is absolutely necessary, we can have an elaborated version of intuition with the features that are very often present, including not only those identified by Sinclair and Ashkanasy or Dane and Pratt, but also other interesting features such as experience and expertise, as proposed by Baldacchino et al. (2015) and Baldacchino (2019). Further studies are needed to assess if these attributes can be put in relation to different types of intuition (creative intuition, expert or problem-solving intuition, etc.). In the emergentist account, all intuition’s attributes can also be observed in the process of insight, well understood in cognitive psychology and social cognition. The differing length of insight and intuition could be due not to differences in the nature of the process (as implied by Dane and Pratt’s distinction) but rather to the quantity of exploration needed to reach a consistency threshold, or, in other words, the degree of recursivity of holistic associations—namely, the number of iterations necessary to reach such a threshold. The wholeness of insight, which fragments under Dane and Pratt’s (2007, p. 40) two-stage conceptualization based on non-conscious incubation and conscious insight, appears better denoted by the emergentist view, entailing an evolutionary path characterized by continuity (iterative holistic associations) and discontinuity (emergence).

## Theoretical and Practical Implications

From a theoretical point of view, the conceptualization of intuition as emergence appears consistent with empirical evidence, particularly relevant for upper echelons. It corroborates in particular the evidence that the intuitive information processing is not necessarily analogous to the actual use of intuitive processes (Blume and Covin, 2011; Baldacchino, 2013) since, in contrast with earlier studies (Sinclair and Ashkanasy, 2005; Evans, 2010), depends on self-organizing processes. Further research is required to explore intuition preferences at the strategic level vs. the actual use of intuitive processes, as well as their relationships with experience and various features of context. Furthermore, the emergentist conceptualization relies on a complexity narrative that, with its capacity to transcend antinomies, can accommodate opposed views in a true Hegelian fashion. In particular, the use of intuition alongside analysis should be explored, to shed light on how these two processing modes interact in shaping upper echelons’ strategies, and to enhance our understanding of dual-process theories.

Moreover, the emergentist account of intuition renders futile the search for a deterministic relationship of intuition effectiveness with static antecedent conditions, such as the complexity of cognitive schemas or the structuredness of tasks. Dane and Pratt model suggests that “cognitive schemas must be domain relevant and complex to generate accurate intuitive judgments” (p. 50), while tasks must be unstructured. This proposition can be enriched by asserting that specificity and complexity of cognitive schemas must be regarded *in relation to* the specific task/environment, in line with our view that intuition emerges from the relationship between the intuiting subject and a set of cues characterizing his/her task/environment. This can be articulated by taking into account the development of behavioral research on decision-making, which focuses on the interplay between the task/environment and the decision-maker, taking the move from the seminal studies of “fast and frugal heuristics” (Gigerenzer et al., 1999), the “adaptive toolbox metaphor” (Gigerenzer and Todd, 1999), and the “adaptive behavior and cognition” (Gaissmaier et al., 2008; Newell and Bröder, 2008; Rieskamp, 2008). According to Gigerenzer, the repertoire of mental shortcuts that human mind uses to arrive at a reasonable judgment, capitalizing on its cognitive limitations as well as on environmental limitations, is not generically effective or ineffective but rather it is ecologically rational, contingent upon its match with the demands of the task and the environment. Consequently, it is insufficient to look separately at persons and tasks, rather it is important to look at their interaction: the level of expertise of a person can be evaluated in relation to a specific task, while the level of structuredness/novelty of a task depends on the decision-maker (Lichtenstein and Slovic, 2006). In other words, we could say that people are experts in particular tasks and not in others, and tasks are complex or emotion-laden for some decision makers and not for others. In this perspective, the “mental model of the task” is the key driver, thus challenging the idea that tasks/environments are objective and stable entities, and building a bridge toward philosophical research. The practical implications are notable: for example, executive team composition should be engineered to match diverse tasks’ expertise with different decision-making scenarios, and career paths should be designed to acquire expertise useful for different decision-making contexts. The phenomenal plurality of intuition outcomes could be explained, too, thus providing suggestions in case of intra-personal inconsistency and possible strategies.

Conceived as emergence, intuition is unpredictable but intelligible, in the sense that it should be possible to identify its evolution pattern. That something might be irreducible does not prevent hypothesizing a theory of how this irreducibility emerges as a consequence of agent interaction: there is an inherent rationale for how the system unfolds; a generative process that transcends the connection of causes and effects. This is another important avenue for research. Barney and Felin (2013, p. 147) recommended that appeal to emergence should not be a means to obfuscate explanation “by hiding the actual mechanisms, processes, and actors that lead to the emergent outcome.” In line with this, the “emergentist” view of intuition should be regarded as a reason to study the phenomenon in depth, seeking—not escaping—scientific explanations.

Such a view opens up to study the use of artificial intelligence in support of managerial decision-making, allowing investigation of whether complex domain-relevant schemes could be transferred to automated information systems, a question posed by Dane and Pratt (2007, p. 49). In fact, it allows a clear distinction between what can be automated (rational processes) and what is irreducible (intuitive processes—emergent, so not reducible). However, this distinction holds *ex ante*. The outcome of intuition cannot be predicted, but once it has emerged, the process can be automatized (transformed into a machine-compatible representation language), since machines can be “trained” and can therefore compactly store and quickly use decision models developed intuitively.

The combination of intuition and AI will be useful when the conditions of intuition’s effectiveness are met, related to the effectiveness of the expertise of the human decision-maker in relation to the properties of the task. As suggested by Vincent (2021, p. 431), “if the decision maker is a novice, it may be prudent to delegate decision-making authority to AI regardless of whether the task is structured or not, since the novice will not have the capacity to supplement or correct a decision derived through extensive computation. Likewise, if the task is structured and an accurate decision can be derived through logical analysis, the decision should be delegated to AI because not even expert humans can match the speed and analytic capabilities of computer systems.”

The challenge is to integrate human and IT resources so as to automate everything automatable, thus freeing human energies for intuition. The equilibrium is dynamic: as the environment evolves, with cues changing or new cues emerging, the automated system should be reassessed. Human intuition is needed for this, and broad, open and vigilant mindfulness is required. So, while advances in IT reduce the use of intuition in the workplace, human beings will always be the guiding force (Sibanda and Ramrathan, 2017).

It is important for future studies to connect the two areas of intuition and information technology, since such research has been quite limited to date (Sibanda and Ramrathan, 2017). Further studies are particularly needed regarding human resources training and development. To ensure that each level of the organization can take full advantage of available IT tools, it is important to study how to reskill the workforce. Knowledge is also required on how to create new roles (such as Chief Analytical Officer or Chief Information Officer) to act as a bridge between business and IT. To avoid deskilling from automation, studies should focus on how to develop human intuition: as Levinthal (2011) observes, it is more important to develop intuition than rationality, since analytic thinking can be supported by automation while intuitive thinking cannot.

The conceptualization of intuition as emergence is also suited to the task of analyzing intuition across levels of analysis, considering in particular the escalation from individual to collective decision-making, which are common dynamics at the basis of management decisions in organizations (Cristofaro, 2019). Although scholars have traditionally explored intuition at the individual level, it may occur at any level of a human organization: it may emerge within a group through the complex interactions among individual intuitions, thus generating a cognitive system that cannot be reduced to the intuitions of individual group members (Huber and Lewis, 2010; Healey et al., 2015). While the findings of research on individual intuition have application to group situations, research on group intuition as a unique phenomenon is quite scarce. In particular, business applications of intuitive processes at the group level have yet to be fully explored.

We need also to explore how individual and group intuition aggregates at the organizational level, including how intrapersonal and interpersonal processes combine to produce emergent phenomena, and how the organization itself, as a social context, affects and shapes individual and group intuitions. As articulated by Heath and Sitkin (2001), cited by Barney and Felin (2013), we need theories of intuition in and of organizations. As reported by Stinchcombe (1990, p. 341) and Barney and Felin (2013) argued that “any theory of organization must explain how organizations can be more rational than individuals (though of course they are not always).” We could enrich this argument by adding “and more intuitive.”

## Conclusion

*“A foreign country is not designed to make you comfortable*…*” C. Fadiman*

To date, the multiplicity and fragmentation of intuition studies, albeit affording much richness and breadth, have resulted in atomistic evolution of research, with few exchanges across disciplines and unrelated groups of researchers, increased replications and redundancies in fieldwork, and limited conceptual integration and knowledge accumulation.

Both Dane and Pratt and Sinclair and Ashkanasy provide a coherent integration of seemingly disparate findings and theories of intuition, bringing order to the anarchy and valuably opening a dialogue with psychology and philosophy. The openness to philosophy is beneficial to allow a better understanding of the historical use as well as the variety of contemporary uses of the concept of intuition but, notwithstanding Dane and Pratt’s claims, no significant dialogue with philosophers has developed, as confirmed by the lack of philosophy scholars commenting on or quoting their work. Dane and Pratt’s authoritative and conceptually appealing statements are widely employed but rarely challenged, risking the perpetuation of contestable assumptions and discouraging efforts toward a richer conceptualization of intuition. After 10 years, a grand theory of human intuition is still lacking.

Standing on the shoulders of giants, we have sought to build on these works by proposing a framework that facilitates interdisciplinary dialogue. Our critical analysis indicates that there is no strong interdisciplinary agreement on Dane and Pratt’s conceptualization, which is far from offering a consolidated philosophical account of intuition.

Notwithstanding the difficulty of finding agreement between distant disciplines such as philosophy and management, we identified two areas of potential agreement—an intuitionist and an anti-intuitionist view of intuition—that can be broadly associated with the two contrasting paradigms of positivism and constructionism. Both views can be considered valuable: the first strengthens intuition’s conceptual framework by reference to its epistemological heritage, thus favoring legitimacy and potential dialogue with important pieces of classic philosophy; the second directs the dialogue to twentieth-century philosophers so as to encompass a post-modern conceptualization of intuition, sensitive to both time and context.

Exploring the possibility of connecting the two contrasting positions without imposing an artificial unity, we conceptualized intuition as emergence. This account of intuition offers a theorization of a different logical type, just like binocular vision: the extra depth offered by three-dimensional vision is of a different logical type to the two-dimensional vision each eye offers to the brain (Bateson, 1979, p. 84).

An important limit of paper is that it addresses just the two definitions by Sinclair and Ashkanasy and by Dane and Pratt. Future scholars can enhance this research by including other points of view. Therefore, it is premature to posit this conceptualization as shared territory for the evolution of intuition theories, and, as shown in the previous paragraph, in-depth studies are needed to evaluate it. However, the paper undoubtedly helps to tackle a multi-faceted challenge. It provides a broader account of intuition, which encompasses relevant cognitive processes such as cold intuitive processes or those (creative, problem-solving, insight) that are contingent on an incubation period, as well as a range of behavioral possibilities other than engaging in conscious thinking or acting immediately upon instant judgments. Besides, it recognizes the interpenetrative and indivisible character of intuitive experience, resisting the overwhelming tendency to spatialize time, which is the dominant, positivist mode of thought governing most studies on intuition, such as that by Dane and Pratt, who conceive intuition and insights as discrete moments deterministically caused by discrete antecedent factors. Finally, with awareness rising that scientific progress is likely to be enhanced from seeking a wider perspective acknowledging the potential contributions of other source disciplines, it can favor fruitful dialogue with scholars from different fields of study, particularly philosophy, thus contributing to the shared cross-disciplinary construction of a grand theory of intuition, like a multi-voice harmony in a jazz ensemble.

There is still a long way to go, and it is not an easy journey for many management scholars, who have been trained to see orderly and linear patterns, lacking the metaphors that allow them to see non-linearity. The difficulty of breaking from positivist views that have dominated our western thinking is evident from the two articles discussed: they embrace a positivist approach that sits uneasily with the epistemology that their own intuition construct implies. Undoubtedly, using emergence as a metaphorical device requires stepping out of the metaphysical comfort of positivism.

## Author Contributions

Both authors wrote, reviewed, and commented on the manuscript, read, and approved the final manuscript.

## Conflict of Interest

The authors declare that the research was conducted in the absence of any commercial or financial relationships that could be construed as a potential conflict of interest.

## Publisher’s Note

All claims expressed in this article are solely those of the authors and do not necessarily represent those of their affiliated organizations, or those of the publisher, the editors and the reviewers. Any product that may be evaluated in this article, or claim that may be made by its manufacturer, is not guaranteed or endorsed by the publisher.
